# Tamarixetin: A Promising Bioflavonoid Against Acetaminophen-Induced Liver Injury

**DOI:** 10.3390/cimb47070524

**Published:** 2025-07-08

**Authors:** Mehmet Ali Telafarlı, Ejder Saylav Bora, Firdes Topal, Oytun Erbaş

**Affiliations:** 1 Department of Emergency Medicine, Erzurum City Hospital, 25240 Erzurum, Turkey; 2 Department of Emergency Medicine, Faculty of Medicine, Izmir Katip Çelebi University, 35360 Izmir, Turkey; 3 Department of Gastroenterology, Faculty of Medicine, Izmir Katip Çelebi University, 35360 Izmir, Turkey; 4 Biruni Research Center (BAMER), Faculty of Medicine, Biruni University, 34015 Istanbul, Turkey

**Keywords:** tamarixetin, acetaminophen, hepatotoxicity, oxidative stress, liver protection

## Abstract

Oxidative stress, mitochondrial dysfunction, and inflammatory responses cause acute liver failure in most cases of acetaminophen (APAP) overdose. Tamarixetin (Trx), an antioxidant and anti-inflammatory flavonoid, has not yet been studied in models of APAP-induced hepatotoxicity. Trx was tested for its protective effects on APAP-induced liver injury in rats using biochemical, histopathological, and oxidative stress parameters. Three groups of 30 male Wistar rats were randomly assigned to the following groups: control, APAP + Saline, and APAP + Trx (3 mg/kg/day, intraperitoneally for 3 days). A single 300 mg/kg intraperitoneal APAP dose caused hepatotoxicity. ALT, MDA, GSH, HSP-70, and thioredoxin were measured in blood and liver tissues. Liver sections were histopathologically examined. APAP depleted hepatic GSH and Trx and increased serum ALT and MDA. Trx treatment significantly reduced ALT (201.2 → 105.1 U/L), MDA (5.5 → 3.4 nmol/mg), and the percentage of histologically damaged hepatocytes (58.5% → 9.5%), while restoring GSH and thioredoxin levels. Notably, HSP-70 expression exceeded that of APAP and control levels, suggesting the modulation of the stress response. The Trx group showed significant hepatoprotection histologically. Trx reduces APAP-induced hepatic damage, likely through antioxidant and anti-inflammatory mechanisms. These findings suggest that Trx may be a natural hepatoprotectant, warranting clinical trials.

## 1. Introduction

N-acetyl-p-aminophenol (APAP), also known as paracetamol, is a pharmaceutical agent widely used for its analgesic and antipyretic properties. Nonetheless, an overdose may result in significant hepatic injury and abrupt hepatic failure [1].

The deleterious effect of APAP is associated with oxidative damage and the production of reactive oxygen species (ROS). APAP undergoes metabolism in the liver via CYP450 enzymes, which is a process that may lead to oxidative stress [1,2,3]. Oxidative stress induces DNA damage and cellular necrosis in hepatocytes [3]. Acetaminophen abuse results in mitochondrial impairment and cellular apoptosis. This process is linked to endoplasmic reticulum stress and inflammatory reactions [4,5].

Diverse antioxidants and botanical substances may effectively mitigate APAP-induced hepatic damage. N-acetyl-cysteine (NAC) mitigates liver damage by restoring cellular glutathione levels [3,6]. Moreover, natural compounds, such as plant polyphenols and flavonoids, confer hepatic protection by mitigating oxidative stress [3]. The activation of molecular pathways, such as Nrf2 and SIRT1, may confer protection against APAP-induced injury. These pathways enhance cellular antioxidant defense mechanisms and diminish inflammatory reactions [2,7].

Tamarixetin (Trx) is a natural flavonoid derivative recognized for its many pharmacological properties. It is a chemical that exhibits anti-inflammatory, antioxidant, antiviral, and anticancer properties. It also exerts cardiovascular effects [8]. Trx alleviates bacterial sepsis by reducing inflammatory responses and promoting the production of anti-inflammatory cytokines, such as IL-10 [9]. These effects were particularly observed in lipopolysaccharide (LPS), which modulate inflammation by inhibiting the activation of the nuclear factor kappa B protein (NF-κB) and the NLRP3 inflammasome [10,11].

This study aimed to demonstrate, for the first time in the literature, the limiting and protective effects of Trx, a potent anti-inflammatory and antioxidant agent, on APAP-induced liver injury in an experimental rat model.

## 2. Materials and Methods

### 2.1. Animals

The study utilized a sample of 30 adult male Wistar rats, averaging 200–210 g in weight. Galenty et al. reported [12] that male rat livers demonstrated a heightened vulnerability to APAP toxicity compared to female livers. Consequently, male animals were selected as the subjects for this study.

During the experiment, the animals were confined in cages and maintained under standard conditions, which included a room temperature of 22 ± 2 °C and a 12 h light/dark cycle. The subjects were provided with potable water and a standard pellet diet ad libitum throughout the study. The Institutional Animal Care and Ethical Committee of the University of Science granted sanction for the study protocol (Ethical Number: 2823111522/16.01.2023). Sigma-Aldrich (Sigma-Aldrich Inc., St. Louis, MO, USA) was the source of all compounds unless otherwise specified.

#### 2.1.1. Experimental Design

**Figure 1:**
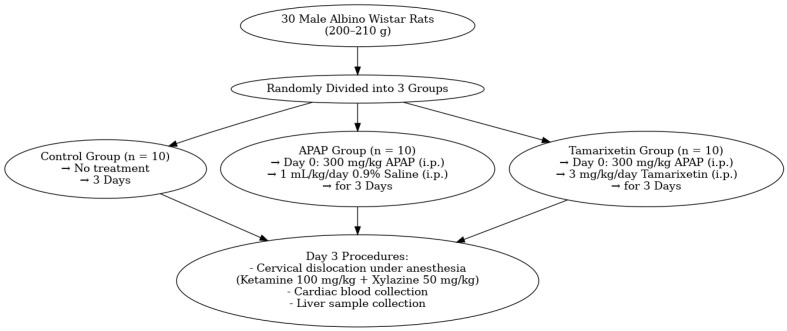
Flowchart of the study.

Thirty male Wistar albino rats were used in the present study. Rats were randomly assigned to three groups. Ten rats were assigned to a normal control group and received no drug. Twenty rats were given a single dose of 300 mg/kg APAP (Parol, Atabay, Istanbul, Turkey 10 mg/mL) intraperitoneally (i.p.). These rats were divided randomly into two groups. Group 1 rats (*n* = 10) (APAP + saline) were given 1 mL/kg/day 0.9% NaCl saline i.p. for three days. Group 2 rats (*n* = 10) (APAP + Tamarixetin) were given 3 mg/kg/day Tamarixetin (Sigma-Aldrich Inc., St. Louis, MO, USA) via i.p. for three days (Figure 1).

After the study, all animals were euthanized via cervical dislocation after anesthesia with ketamine (100 mg/kg, Ketasol, Richterpharma AG, Wels, Austria) and xylazine (50 mg/kg, Rompun, Bayer, Germany). Blood samples were obtained through a heart puncture for biochemical analysis. Liver specimens were collected for histopathological and biochemical analysis.

#### 2.1.2. Histopathological Studies of the Liver

The liver was removed and fixed for 3 days in 10% formaldehyde in 0.1 M phosphate-buffered saline (PBS). Formalin-fixed liver sections (4 μm) were stained with hematoxylin and eosin. All sections were photographed with an Olympus C-5050 digital camera mounted on an Olympus BX51 microscope (Shinjuku, Tokyo, Japan).

The morphological evaluation of the liver was performed using a computerized image analysis system (Image-Pro Express 1.4.5, Media Cybernetics, Inc., Rockville, MD, USA) on 10 microscopic fields per section, examined at a magnification of ×40 by an observer who was blind to the study group. The rate of damaged hepatocytes was measured as a percentage of the total number of hepatocytes.

#### 2.1.3. Assessment of Plasma ALT Concentration

The enzyme-linked immunosorbent assay (ELISA) kit, commercially available from USCN Life Science Inc. (Wuhan, China), was used to determine the levels of ALT in the plasma.

#### 2.1.4. The Biochemical Analysis of the Liver

After being sacrificed, the liver was instantly removed and stored at a temperature of −20 degrees Celsius until the biochemical test was performed. Following the homogenization of the livers using a glass homogenizer in five liters of phosphate-buffered saline (pH 7.4), the livers were centrifuged at 5000× *g* for fifteen min. Following this, the supernatant was collected, and the total protein content in the homogenates was determined by employing Bradford’s method, with bovine serum albumin serving as the standard [13].

We used commercially available rat enzyme-linked immunosorbent assay (ELISA) kits to determine the quantities of HSP-70 and Trxin present in the supernatants. The manufacturer’s specifications were followed in order to ensure that every sample from each animal was measured independently. To determine these absorbances, a microplate reader (manufactured by Thermo Fisher Scientific, located in Waltham, MA, USA) was used.

#### 2.1.5. Assessment of Hepatic Lipid Peroxidation

The amounts of malondialdehyde (MDA), as a thiobarbituric acid-reactive substance (TBARS), were measured in tissue samples to estimate the level of lipid peroxidation [13]. After adding trichloroacetic acid and the TBARS reagent to the tissue samples, the samples were mixed and then incubated at 100 °C for 60 min. To determine the absorbance of the supernatant, the samples were centrifuged at 3000× *g* revolutions per minute for 20 min after being cooled on ice. The levels of MDA were determined using tetraethoxypropane to generate a standard calibration curve, and the results were reported as nanomoles per milligram of protein [14].

#### 2.1.6. Assessment of Hepatic Glutathione (GSH) Concentrations

Using Ellman’s approach [15], spectrophotometric analysis was performed on liver samples in order to determine the amount of GSH present. This technique involves the interaction of thiols with 5,5′-dithiobis (2-nitrobenzoic acid) (DTNB), resulting in a colored anion with a peak at 412 nm. GSH concentrations were determined using a standard calibration curve, and the results were reported as nanomoles per milligram of protein [15].

### 2.2. Statistical Analysis

IBM SPSS 15.0 was employed to analyze the data. The statistics of variables are displayed as the mean plus or minus the standard error of the mean (SEM). The distribution of the numerical variable was determined using the Shapiro–Wilk normality test. The independent sample *t*-test was employed for regularly distributed data, while the Mann–Whitney U test was applied for non-normally distributed data to compare the two groups. A *p*-value below 0.05 was deemed statistically significant.

## 3. Results

### 3.1. Liver Enzymes

Serum ALT levels were markedly increased in the APAP + saline group (201.2 ± 6.7 U/L) relative to the normal controls (41.4 ± 2.6 U/L; *p* < 0.001). Rats administered 3 mg/kg/day of Tamarixetin exhibited a significant reduction in ALT levels (105.1 ± 9.8 U/L) compared to the APAP group (*p* < 0.001). However, these values remained elevated compared to the control group (*p* < 0.01) (Table 1).

### 3.2. Oxidative Stress Markers

APAP exposure resulted in a substantial elevation in hepatic MDA levels (5.5 ± 0.2 nmol/mg protein) compared to the control group (1.16 ± 0.1 nmol/mg protein; *p* < 0.01), indicating increased lipid peroxidation. Tamarixetin administration markedly decreased MDA levels to 3.4 ± 0.1 (*p* < 0.05 compared to APAP), although it did not restore them to baseline levels.

Correspondingly, hepatic GSH levels, which were markedly depleted by APAP (1.8 ± 0.1 vs. 6.09 ± 0.3 in control; *p* < 0.001), were significantly restored by Tamarixetin (5.3 ± 0.2; *p* < 0.001 vs. APAP), approaching normal levels (Table 1).

### 3.3. Redox Regulation and Stress Proteins

APAP markedly reduced liver thioredoxin (Trx) levels (8.3 ± 0.5 pg/mg) in comparison to the control group (15.8 ± 1.3; *p* < 0.001). Tamarixetin treatment enhanced Trx expression (12.9 ± 0.8; *p* < 0.001 vs. APAP), yet it remained marginally below the control values (*p* < 0.05).

The expression of HSP-70 exhibited a modest increase in the APAP group (9.2 ± 0.2 pg/mg) compared to the control group (7.5 ± 0.1; *p* < 0.05). Tamarixetin significantly increased HSP-70 levels (14.8 ± 0.9), surpassing both the APAP group (*p* < 0.05) and control animals (*p* < 0.001) (Table 1).

### 3.4. Histopathological Damage

**Figure 2:**
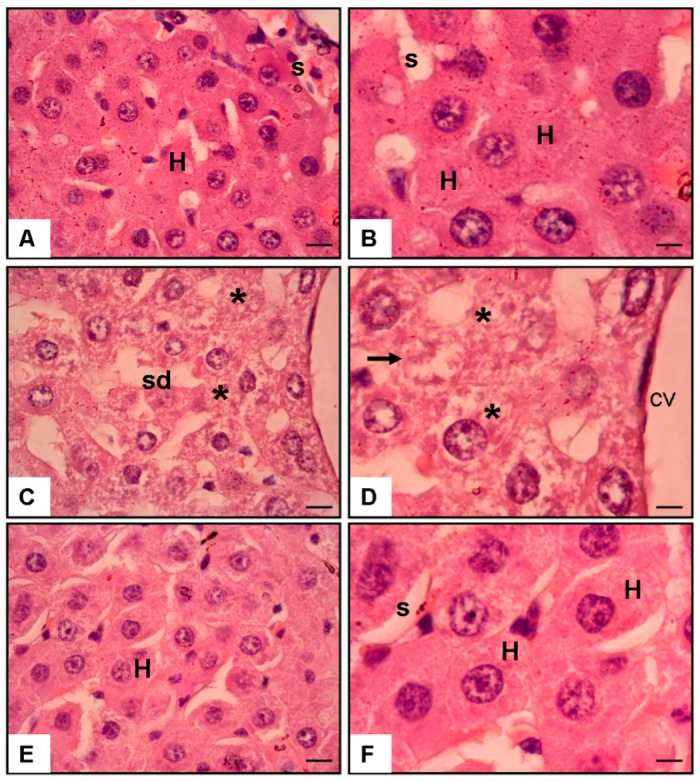
The hematoxylin and eosin (H & E) staining of rat liver sections at 40× and 100× magnification. (**A**,**B**) Normal liver tissue exhibits sinusoids (s) and hepatocytes (H). (**C**,**D**) The liver from the APAP and saline group showed cellular injury (asterisks), necrosis (arrows), and sinusoidal dilatation (sd) in the centrilobular vein (cv). (**E**,**F**) The liver from the APAP and 3 mg/kg/day Tamarixetin group demonstrated no cellular injury or necrosis in the centrilobular area (scale bar = 200 μm).

The proportion of damaged hepatocytes rose sharply following APAP exposure (58.5 ± 2.6%) compared to normal liver tissue (4.2 ± 0.1%; *p* < 0.001). Tamarixetin treatment resulted in a substantial reduction in hepatocellular damage (9.5 ± 0.8%; *p* < 0.001 vs. APAP), with values approaching those of the control group (*p* < 0.05) (Table 1, Figure 2).

### 3.5. Histological Findings (Figure 2)

Microscopic analysis corroborated the biochemical results. The control liver tissue displayed intact hepatic cords and sinusoids. In contrast, APAP exposure caused prominent centrilobular necrosis, sinusoidal dilatation, and cellular disruption. Tamarixetin-treated liver sections showed the preservation of hepatic architecture with minimal signs of necrosis or inflammatory infiltration.

## 4. Discussion

The first 8 h are critical in APAP poisoning. Despite experimental studies [16,17], no accessible, inexpensive, and effective agent other than N-acetylcysteine (NAC) has been found in the current literature.

In a study by Lijaz et al. [18] El-Aarag et al. [19], Tamarixetin has been shown to significantly reduce MDA levels in the liver, which are typically elevated due to oxidative stress. This decrease signifies its antioxidant capabilities, which aid in alleviating the liver damage caused by environmental toxins such as polystyrene microplastics and carbon tetrachloride (CCl4). In examining the mechanism of action of Tamarixetin, it is proposed that this flavonoid enhances the activity of antioxidant enzymes, including superoxide dismutase, catalase, and glutathione peroxidase, which collectively neutralize reactive oxygen species (ROS) and diminish oxidative stress markers, such as malondialdehyde (MDA). Similarly, this study found that the Tamarixetin group had reduced liver MDA levels. Tamarixetin specifically aids in reestablishing the equilibrium of pro- and anti-apoptotic proteins, consequently diminishing cellular mortality and tissue injury [19].

GSH, an essential antioxidant in the liver, plays a pivotal role in sustaining liver function and protecting against hepatotoxicity [20]. The level of glutathione is pivotal in the pathogenesis of liver disease, and its modulation may provide therapeutic advantages for numerous liver disorders [21]. Variations in GSH levels are intricately associated with the pathogenesis of multiple liver diseases, such as alcoholic liver disease and nonalcoholic fatty liver disease [21]. Tamarixetin’s capacity to sustain GSH levels underscores its potential to promote liver health and avert liver diseases [18,20]. In this study, the GSH level is expected, based on other studies, to reach a normal level. By interpreting MDA and GSH levels, we can observe that Tamarixetin exhibits antioxidative properties for the liver.

HSP-70 exhibits hepatic cellular properties, functioning as a protective protein that maintains cellular homeostasis in response to diverse stressors. HSP-70 is upregulated in hepatic cells in response to oxidative stress, such as that induced by APAP. HSP-70 offers cytoprotection by diminishing cytotoxicity and oxidative injury [22]. In a study by Omar et al., they indicated that HSP-70 may play a role in the pathogenesis of alcoholic liver disease and act as a more sensitive marker of hepatocellular injury [23]. Moreover, in ischemic reperfusion injury [24] and in surgical trauma [25], HSP-70 plays similar roles in the liver. In this study, APAP provokes the HSP-70 while damage occurs in hepatocytes; however, Tamerixetin is more likely to induce it, possibly accelerating the healing process.

Trx is a stress-inducible protein that significantly contributes to the regulation of oxidative stress, a prevalent factor in liver diseases such as nonalcoholic steatohepatitis (NASH) and hepatitis C virus (HCV) infection. Increased serum Trx levels have been noted in patients with NASH- and HCV-related liver diseases, suggesting its potential as a biomarker for oxidative stress and disease severity [26,27,28]. Moreover, drug-induced liver injury, exemplified by APAP, disrupts the Trx and glutathione (GSH) systems, resulting in heightened oxidative stress and hepatic damage [29]. Dietary selenium concentrations may impact the severity of APAP-induced hepatotoxicity by modulating the Trx system [30]. Arsenic exposure similarly elevates Trx expression, potentially functioning as a protective mechanism against arsenic-induced hepatic injury by mitigating oxidative stress [31].

On the other hand, a study by Xu et al. found that Thioredoxin-interacting proteins (TXNIPs) are recognized as a crucial factor in mitochondrial dysfunction and oxidative stress, particularly in response to endocrine-disrupting chemicals such as di (2-ethylhexyl) phthalate (DEHP). The upregulation of TXNIP intensifies oxidative stress and inflammation, leading to hepatic injury [32]. The changes in liver thioredoxin levels observed in this study, in conjunction with the results of MDA, HSP-70, and GSH levels, further strengthened the evidence of the antioxidant and anti-inflammatory effects of Tamarixetine.

Histopathological analysis demonstrated that Tamarixetin can markedly diminish liver damage. In investigations of liver injury induced by polystyrene microplastics, Tamarixetin administration resulted in a significant improvement in liver histology, diminishing necrotic and apoptotic alterations in hepatic tissues, as observed in the study by Kayalı et al. with sinomenine administration after APAP toxicity [18,33]. Moreover, the regression in ALT level suggests its potential as a therapeutic agent in alleviating liver damage through its antioxidant and anti-inflammatory properties, as observed in this histopathological analysis.

Tamarixetin is 4′-O-methylquercetin, differing from quercetin by a single methyl group. This minor structural change significantly alters its biochemical behavior, including metal chelation, antioxidant activity, and thiol reactivity [34,35]. Quercetin has been studied more extensively than Trx in terms of hepatotoxicity and has been found to have antioxidant [36,37,38] and anti-inflammatory effects [37,39]. Moreover, in a study by Li et al., Trx was described as more bioavailable than quercetin after oral administration and less thiol-toxic than quercetin [40].

This study has some limitations. The study was conducted on a limited number of rats, and only male animals were used; therefore, the results may not fully reflect those of other populations. A 3-day treatment period was selected to evaluate the acute hepatoprotective effects following APAP-induced injury; however, long-term effects and different dosing regimens were not investigated. Additionally, the molecular mechanisms were not thoroughly explored, and comparisons with standard treatments, such as N-acetylcysteine, were not conducted.

## 5. Conclusions

This experimental study demonstrated that Tamarixetin mitigates liver damage induced by high doses of paracetamol in rats. Enhancements were noted in both biochemical indicators and histological assessments. The results indicate that Tamarixetin may confer protective effects against hepatic injury. Nevertheless, further research is needed to elucidate its mechanisms, assess its efficacy, and evaluate its safety across various conditions.

## Figures and Tables

**Table 1:** Biochemical and histopathological parameters in liver tissues across experimental groups. Values are expressed as mean ± SEM * *p* < 0.05 and ** *p* < 0.001 (different from control); # *p* < 0.05; and ## *p* < 0.001 (different from APAP + saline). ALT: Alanine aminotransferase; MDA: malondialdehyde; GSH: glutathione; HSP-70: heat shock protein 70.

	Normal Group	APAP and Saline	APAP and 3 mg/kg/day Tamarixetin
ALT (U/L)	41.4 ± 2.6	201.2 ± 6.7 **	105.1 ± 9.8 ##
Liver MDA Level (nmol/mg protein)	1.16 ± 0.1	5.5 ± 0.2 *	3.4 ± 0.1 #
Liver GSH Level (nmol/mg protein)	6.09 ± 0.3	1.8 ± 0.1 **	5.3 ± 0.2 ##
Liver HSP-70 Level (pg/mg protein)	7.5 ± 0.1	9.2 ± 0.2 *	14.8 ± 0.9 #
Liver ThioredoxinLevel (pg/mg protein)	15.8 ± 1.3	8.3 ± 0.5 **	12.9 ± 0.8 ##
Damaged Hepatocytes (percentage)	4.2 ± 0.1	58.5 ± 2.6 **	9.5 ± 0.8 ##

## Data Availability

The data supporting the findings of this study are available from the corresponding author upon reasonable request.

## Abbreviations

HSP-70	Heat shock protein 70
MDA	Malondialdehyde
Trx	Tamarixetin
APAP	N-acetyl-para-aminophenol
PBS	Phosphate-buffer saline
ELISA	Enzyme-linked immunosorbent assay
